# Impact of hepatic steatosis on treatment response of autoimmune hepatitis: A retrospective multicentre analysis

**DOI:** 10.3389/fimmu.2022.1040029

**Published:** 2022-12-14

**Authors:** Peiyan Liu, Mingkai Li, Lili Zhao, Hongsheng Yu, Chang Zhao, Jianning Chen, Ruifang Shi, Li Zhou, Qi Zhou, Bin Wu, Jia Li

**Affiliations:** ^1^ Clinical School of the Second People’s Hospital, Tianjin Medical University, Tianjin, China; ^2^ Department of Hepatology, Tianjin Second People’s Hospital, Tianjin, China; ^3^ Department of Gastroenterology, The Third Affiliated Hospital of Sun Yat-Sen University, Guangzhou, China; ^4^ Guangdong Provincial Key Laboratory of Liver Disease Research, The Third Affiliated Hospital of Sun Yat-Sen University, Guangzhou, China; ^5^ Department of Pathology, The Lingnan Hospital of Sun Yat-Sen University, Guangzhou, China; ^6^ Department of Pathology, The Third Affiliated Hospital of Sun Yat-Sen University, Guangzhou, China; ^7^ Department of Pathology, Tianjin Second People’s Hospital, Tianjin, China

**Keywords:** AIH, hepatic steatosis, controlled attenuation parameter, treatment response, risk, prognosis

## Abstract

**Background:**

There is a paucity of data on whether steatosis impacts autoimmune hepatitis (AIH) treatment response. We aimed to evaluate the influence of baseline steatosis on the biochemical response, fibrosis progression, and adverse longterm outcomes of AIH.

**Methods:**

Steatosis was diagnosed by a controlled attenuation parameter (CAP) ≥ 248 dB / m. Only patients who underwent immunosuppressive therapy with available liver histological material at diagnosis and qualified CAP within seven days of the liver biopsy were included. Univariate and multivariate analyses were subsequently conducted.

**Results:**

The multicentre and retrospective cohort enrolled 222 subjects (88.3% female, median age 54 years, median follow-up 48 months) in the final analysis, and 56 (25.2%) patients had hepatic steatosis. Diabetes, hypertension, and significant fibrosis at baseline were more common in the steatosis group than in the no steatosis group. After adjusting for confounding factors, hepatic steatosis was an independent predictor of insufficient biochemical response (OR: 8.07) and identified as an independent predictor of long-term adverse outcomes (HR: 4.07). By subgroup multivariate analysis (different degrees of steatosis, fibrosis, and prednisone dose), hepatic steatosis independently showed a relatively stable correlation with treatment response. Furthermore, in contrast to those without steatosis, a significant increase in liver stiffness (LS) was observed in patients with steatosis (4.1%/year vs. -16%/year, P < 0.001).

**Conclusions:**

Concomitant hepatic steatosis was significantly associated with poor response to treatment in AIH patients. Routine CAP measurements are therefore essential to guide the management of AIH.

## Introduction

Nonalcoholic fatty liver disease (NAFLD) is rapidly becoming the most common cause of chronic liver disease worldwide (almost 25% of the general population (1)). It can progress to cirrhotic complications and hepatocellular carcinoma (HCC) 20 years after diagnosis (2). Hepatic steatosis triggers lipotoxicity, oxidative stress, and inflammation, such as Treg dysfunction and increased cytokine (IFN-γ, IL-17A) production by activated Th1, Th2, Th9, and Th17 cells, which can aggravate impaired immune regulation, presenting abnormal ANA at rates of 16-34% (3–5).

Several studies have demonstrated that NAFLD frequently coexists with other liver diseases, such as chronic viral hepatitis, alcohol-related liver disease, and autoimmune hepatitis (AIH), which might cause worse clinical outcomes and poorer survival than single chronic liver disease (6). However, there is little knowledge about the consequences of AIH and NAFLD coexistence.

Evidence suggests that the prevalence of both hepatic steatosis and AIH is 17-30% in adult patients (7–10). Concomitant hepatic steatosis might harm clinical outcomes for several reasons: 1) The results of a single centre recruiting a small number of patients predicted that concomitant hepatic steatosis would be a relative risk factor for liver-related adverse outcomes (8). 2) A mouse model concluded that preexisting NAFLD could potentiate the severity of AIH by elevating the numbers of liver autoantigen-specific T cells (11). 3) Steatosis is related to increased insulin resistance and advanced liver fibrosis. 4) The pathogenesis is similar for both AIH and NAFLD. CD4^+^ T cells play a vital role, and increased immune reactivity of NAFLD could therefore aggravate the immune-mediated imbalance of AIH (12). Therefore, the cooccurrence of AIH and hepatic steatosis requires close, lifelong follow-up.

The American Association for the Study of Liver Diseases (AASLD) practice guidance of AIH in 2019 also indicated that concurrent NAFLD might influence the response to therapy (10). However, there is insufficient evidence about the effects of hepatic steatosis on continuous remission or relapse. When concomitant AIH and NAFLD occur, there is a lack of data reporting the impact of NAFLD on the treatment response of AIH.

There is a significant clinical advantage in promoting noninvasive over invasive methods. At present, the study of fat infiltration in AIH patients mainly relies on pathological biopsy. Due to the uneven distribution of steatosis throughout the liver parenchyma, there may be sampling errors and inaccurate staging in assessment by liver biopsy (13). Notably, the controlled attenuation parameter (CAP) can be used as a noninvasive quantitative diagnostic method to remove subjectivity (14) and with well-defined cut-offs applied for different degrees of steatosis (15). A recent analysis confirmed that CAP can effectively evaluate hepatic steatosis in patients with autoimmune liver disease (AILD), regardless of the acute or chronic course (16). CAP can identify steatosis independently of intrahepatic inflammation (such as ALT elevation) (17–19). This paper includes multicentre research data with a relatively large sample size to analyse the impact of baseline hepatic steatosis on the biochemical response, development of liver fibrosis, and long-term clinical outcome of AIH patients by innovatively utilizing this easily accessible steatosis quantification. Furthermore, a study showed that biochemical response is a reliable surrogate marker, at least as good as histological remission (20). This study innovatively applied biochemical response rather than histological remission to assess treatment response.

## Materials and methods

### Patient collections

**Figure 1:**
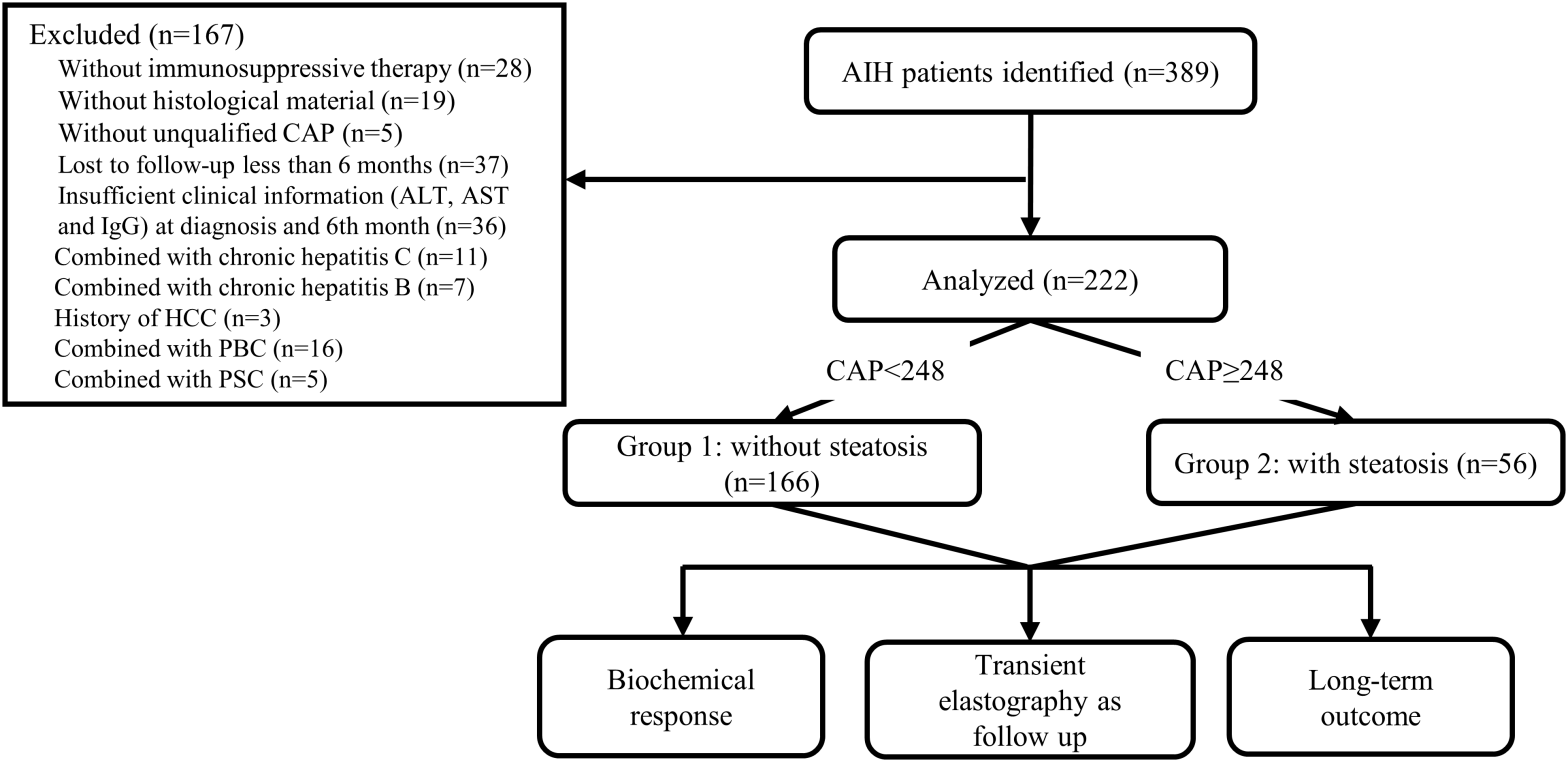
Flowchart of the study. AIH autoimmune hepatitis, CAP controlled attenuation parameter, ALT alanine aminotransferase, AST aspartate aminotransferase, IgG immunoglobulin G, HCC hepatocellular carcinoma, PBC primary biliary cirrhosis, PSC primary sclerosing cholangitis.

This multicentre, retrospective study involved AIH patients from three centres in China between January 2011 and December 2021. Patients with a pretreatment International Autoimmune Hepatitis Group (IAIHG) score ≥ 10 or a simplified score ≥ 6 were identified by searching the medical records database. Only patients who underwent immunosuppressive therapy with available liver histological material at diagnosis and qualified CAP within seven days of the liver biopsy were included. Patients were excluded if they had other causes of liver disease, including viral hepatitis, primary biliary cholangitis, primary sclerosing cholangitis, autoimmune overlap syndromes, drug-induced liver disease, alcoholic liver disease (≥ 30 g per day for males or ≥ 20 g per day for females), Wilson’s disease, hereditary haemochromatosis, and HCC through careful collection and a full serological screen. Patients were excluded if the follow-up period did not exceed six months or they lacked available ALT, AST, and IgG at diagnosis and at sixth months. In this study, 222 subjects were recruited, and the flow of all patient enrolment is shown in **Figure 1**. The study protocol was approved by the Ethics Committees of three participating centres and adhered to the principles of the Declaration of Helsinki. Written informed consent was obtained from each patient.

### Clinical and laboratory assessment

Available baseline demographic data, medical history, and laboratory test variables were derived from medical records. The diagnosis of liver cirrhosis was primarily based on clinical imaging and symptoms. All blood samples were collected at the same time as diagnostic biopsy, between 8:00 and 11:00 am, after an overnight fast (≥12 hours). In three hospitals, the upper limits of normal (ULN) for alanine aminotransferase (ALT) were 50 U/L, 35 U/L, and 40 U/L, and those for aspartate aminotransferase (AST) were 40 U/L, 35 U/L, and 40 U/L. The ULN for immunoglobulin G (IgG) was 16 g/L at the three hospitals. ULNs for other variables are attached in the footnotes of each table. All patients were also tested for the following antibodies by indirect immunofluorescence with an initial dilution concentration of 1:100: antinuclear antibody (ANA), smooth muscle antibody (SMA), antimitochondrial antibody (AMA), liver-kidney microsomal-1 antibody (LKM-1) and soluble liver antigen/liver pancreas antibody (SLA/LP).

### Liver histology

All histological materials at diagnosis were available in this cohort. The typical histological feature comprises interface hepatitis with lymphoplasmacytic or lymphocytic infiltration, emperipolesis, and hepatic rosette formation. Experienced hepatopathologists reviewed all liver tissue specimens without reference to clinical details. Any disagreements were discussed between centres. Pathology was reviewed again by an experienced pathology director (RS) before enrolment. The histological features, including interfacial inflammation (A0-A4, significant inflammation: A3-A4, no significant inflammation: A0-A2), plasma cell infiltration, and rosettes, were recorded according to the Ishak grading scheme (21). The Batts–Ludwig scoring system was also used to assess the fibrosis stages (F0-F4) (22, 23). F0-F2 was defined as no significant fibrosis, and the F3-F4 stage was defined as significant fibrosis. The infiltration extent of fatty acids was also recorded at low magnification. Hepatic steatosis was graded from 0 to 3: S0 < 5%, S1 = 5-33%, S2 = 34-66%, and S3 > 67% (24). Nonalcoholic fatty liver disease activity scores (NAS) were assessed for overall histological change as previously reported on a scale of 0-8 [sum of scores for hepatic steatosis (0-3), lobular inflammation (0-3), and hepatocyte ballooning (0-2)] (25).

### TE measurement

After more than a 6-hour fast, CAP and liver stiffness (LS) measurements were performed using the FibroScan 502 machine (Echosens^®^, Paris, France). The M probe was used for patients with BMI < 30 kg/m^2^, while the XL probe was used for patients with BMI ≥ 30 kg/m^2^. At least 500 transient elastography procedures were performed by four certified operators with prior formal training from Echosens^®^. CAP was considered poorly reliable, with an interquartile range of ≥ 40 dB/m (26). The diagnosis of hepatic steatosis was defined as CAP ≥ 248 dB/m (mild steatosis: 248-267 dB/m, significant steatosis: ≥ 268 dB/m) (15, 27), and LS was used to quantify hepatic fibrosis. Measurements were performed at least ten times, with a success rate of ≥60% and a median/IQR ratio of < 30% considered reliable (28). All CAP and LS values of enrolled patients were proven reliable. We selected CAP within seven days of diagnostic liver biopsy as the baseline level. We recorded baseline LS approximately six months after therapy to avoid potential bias due to hepatic inflammation. It was expressed as the mean value if conducted multiple times a year.

### Treatment and study definitions

Our treatment followed the AASLD guidelines of AIH (10). Patients received corticosteroids starting at an oral dosage of 10-60 mg/day that was tapered gradually to a maintenance dosage of 5-10 mg/day. Additionally, azathioprine (AZA) was given as a treatment compound at a 50 mg/day dose. If patients were intolerant or unresponsive to corticosteroids or AZA, mycophenolate mofetil (MMF) was recommended to add corticosteroids or replace AZA. According to the recent IAIHG research (29), the complete biochemical response was defined as normalizing serum transaminases and IgG within six months of initial treatment. An insufficient response was defined as a lack of complete biochemical response with abnormal serum AST, ALT, or IgG levels within six months. Nonresponse was defined as a < 50% decrease in serum transaminases within four weeks after initiation of treatment. Adverse outcomes included liver cirrhosis (no primary cirrhosis), decompensated liver cirrhosis (manifested as portal hypertension, variceal bleeding, ascites, or hepatic encephalopathy), liver-related death, liver transplantation, and HCC. Clinical data were assessed every six months. The follow‐up time was calculated from the date of treatment initiation until the last visit to the outpatient clinic, liver‐related death, or liver transplantation.

### Statistical analysis

All continuous variables are expressed as the means (± standard deviation) or as the medians (quartile 25, quartile 75), depending on whether they were normally distributed. Differences were compared using Student’s t test, Mann‐Whitney U test, or Kruskal‐Wallis test. Categorical variables are expressed as the frequencies (n) and percentages (%). The chi-squared test or Fisher’s exact test was applied to compare categorical variables.

Univariate and multivariate analyses were carried out to assess the impact of hepatic steatosis at baseline on biochemical response and adverse outcome with a logistic regression model and Cox proportional hazard model, respectively. To minimize the risk of a type I error, we only investigated readily available variables and had a plausible pathophysiological link to the outcome of interest. Odds ratios (ORs), hazard ratios (HRs), and 95% confidence intervals (CIs) were calculated. To remove the influence of confounding factors, we selected confounders based on prior knowledge and adjusted them. Model 1 was adjusted for sex, age, BMI, arterial hypertension, diabetes mellitus, and fasting plasma glucose (FBG). Model 2 was further adjusted for ALT, AST, IgG, cirrhosis, fibrosis, interface hepatitis, biochemical response, and corticosteroid dose. We also conducted sensitivity analyses targeting the effect of hepatic steatosis with a multivariate regression model by performing subgroup analyses (degree of steatosis, fibrosis, and corticosteroid dose).

A generalized mixed model (intercept: none; slope: average changes in LS per year as percentages from baseline values) was used for different statuses of hepatic steatosis, biochemical remission, and long-term prognosis to assess the progression rate of LS and its 95% CI. Clinical variables and time were considered fixed and random effects, respectively. Because the degree of LS change (△LS) was not uniform every year, we also calculated the △LS in the first, second, and third years. △LS% was calculated as a percentage from baseline values.

Statistical analysis was performed with SPSS version 25.0 (IBM, Armonk, NY, USA), Prism 8.0 (GraphPad Software, San Diego, CA, USA), and R software (version 4.2.1, http://www.R-project.org). All tests were two-tailed, and a P value < 0.05 was regarded as significant.

## Results

### Baseline characteristics

In the final analysis, two hundred twenty-two patients with reliable TE and satisfactory liver biopsy specimens were recruited. A total of 88.3% of the whole cohort was female, and the median age at diagnosis was 54 (43, 62). The serum ALT, AST, and IgG levels in the ULN were 4.18 (1.42, 9.35), 4.21 (1.57, 8.86), and 1.17 (0.91, 1.56), respectively. At least one antibody (ANA, SMA, SLA/LP, and anti-LKM-1) was positive. Moreover, significant fibrosis was present in 97 (43.7%) patients. Further baseline characteristics are given in **Table 1**. According to the histological assessment, the boxplot of CAP *vs.* steatosis grade is shown in **Supplementary Figure 1A**. CAP significantly differed among S0-S3 (Kruskal‐Wallis test P < 10^-4^). For CAP *vs.* NAS grade distribution, CAP was also significantly different (Kruskal‐Wallis test P < 10^-4^) (**Supplementary Figure 1B**).

**Table 1:** Clinical, biochemical, immunological and histological features of patients at baseline.

Variables	Data (n=222)
Clinical features	
Female gender, n (%)	196 (88.3)
Age at diagnosis, y	54 (43, 62)
BMI (kg/m^2^)	23.01 ± 3.16
CAP (dB/m)	210.6 ± 44.03
Steatosis based on CAP, n (%)	56 (25.2)
Arterial hypertension, n (%)	44 (19.8)
Diabetes mellitus, n (%)	31 (14.0)
Extrahepatic autoimmune disease, n (%)	44 (19.8)
Cirrhosis, n (%)	53 (23.9)
AIH revised diagnosis score	16 (13, 18)
AIH simplified diagnosis score	7 (6, 8)
Laboratory values at diagnostic biopsy	
PLT/ULN	0.52 ± 0.21
ALT/ULN	4.18 (1.42, 9.35)
AST/ULN	4.21 (1.57, 8.86)
GGT/ULN	2.6 (1.29, 4.48)
ALP/ULN	0.95 (0.72, 1.27)
Albumin/ULN	0.74 (0.67, 0.81)
TBIL/ULN	1.17 (0.62, 3.50)
FBG/ULN	0.86 (0.76, 1.00)
TGs/ULN	0.73 (0.54, 1.12)
TC/ULN	0.71 ± 0.24
Serum immunological features	
IgG/ULN	1.17 (0.91, 1.56)
ANA +, n (%)	209 (94.1)
SMA +, n (%)	103 (46.4)
SLA/LP +, n (%)	11 (5.0)
Anti-LKM-1 +, n (%)	17 (7.7)
AMA +, n (%)	3 (1.4)
Histological features at diagnostic biopsy	
Steatosis, n (%)	43 (19.4)
S0	7 (3.2)
S1	26 (11.7)
S2	7 (3.2)
S 3	3 (1.3)
NAS, n (%)	
<3	24 (10.8)
3/4	7 (3.2)
>4	12 (5.4)
Interface hepatitis, n (%)	
No significant hepatitis ^a^, n (%)	139 (62.6)
Significant hepatitis ^a^, n (%)	83 (37.4)
Hepatocyte resetting, n (%)	54 (24.3)
Plasma cells, n (%)	157 (70.7)
Fibrosis, n (%)	
No significant fibrosis ^b^, n (%)	125 (56.3)
Significant fibrosis ^b^, n (%)	97 (43.7)

Data are presented as the medians (quartile 25, quartile 75)/means (± standard deviation) or numbers (proportion).

a No significant inflammation: A0-A2, significant inflammation: A3-A4.

b No significant fibrosis: F0-F2, significant fibrosis: F3-F4.

ULN values: PLT 350×10^9^/L, ALT 35-50 U/L, AST 35-40 U/L, GGT 45-60 U/L, ALP 125-135 U/L, albumin 51-55 g/L, TBIL 20-23.9 μmol/L, FBG 6.1 mmol/L, TG 1.7-1.92 mmol/L, TC 5.18-5.7 mmol/L, IgG 16 g/L.

ULN upper limits of normal, AIH autoimmune hepatitis, BMI body mass index, CAP controlled attenuation parameter, PLT platelet, ALT alanine aminotransferase, AST aspartate aminotransferase, ALP alkaline phosphatase, GGT gamma-glutamyl transpeptidase, TBIL total bilirubin, FBG fasting blood glucose, TGs triglycerides, TC total cholesterol, IgG immunoglobulin G, ANA antinuclear antibody, SMA smooth muscle antibody, SLA/LP soluble liver antigen/liver pancreas antibody, LKM-1 liver-kidney microsomal-1 antibody, AMA antimitochondrial antibody, NAS nonalcoholic fatty liver disease activity scores.

### Different baseline characteristics with/without hepatic steatosis

Patients were divided into two groups according to CAP values: CAP < 248 for no hepatic steatosis (n = 166, 74.8%) and CAP ≥ 248 for hepatic steatosis (n = 56, 25.2%). The baseline clinical, biochemical, immunological, and pathological characteristics of the two groups are compared in **Table 2**. Overall, there was no significant difference in age (53 *vs.* 56 years, P = 0.399), sex distribution (females: 90.4% *vs.* 82.1%, P = 0.098), or percentage of cirrhosis (22.3% *vs.* 28.6%, P = 0.34). Diabetes (37.5% *vs.* 6%, P < 0.001) and hypertension (41.1% *vs.* 12.7%, P < 0.001) were more common in patients with hepatic steatosis. Patients with hepatic steatosis at diagnosis had higher BMI (24.98 *vs.* 22.13, P < 0.001) and IgG (1.29 *vs.* 1.09 for ULN, P =0.009), although ALT (3.33 *vs.* 4.54 for ULN, P = 0.214) and AST (3 *vs.* 4.6 for ULN, P = 0.324) were lower but not significantly lower. Notably, 36 (64.3%) patients with steatosis had a higher proportion of significant fibrosis stage (P < 0.001), although cirrhosis was not significantly different.

**Table 2:** Baseline clinical, biochemical, immunological and histological features of AIH patients based on hepatic steatosis status.

Variables		AIH only (n=166)	AIH with steatosis (n=56)	*P*
Clinical features				
Female gender, n (%)		150 (90.4)	46 (82.1%)	0.098
Age at diagnosis, y		53 (42, 62)	56 (48, 62)	0.399
BMI (kg/m^2^)		22.13 ± 2.89	24.98 ± 2.86	<0.001
CAP (dB/m)		192.01 ± 33.59	265.68 ± 16.51	<0.001
Arterial hypertension, n (%)		21 (12.7)	23 (41.1)	<0.001
Diabetes mellitus, n (%)		10 (6)	21 (37.5)	<0.001
Extrahepatic autoimmune disease, n (%)		34 (20.5)	10 (17.9)	0.670
Cirrhosis, n (%)		37 (22.3)	16 (28.6)	0.340
AIH revised diagnosis score		16 (13, 18)	16 (14, 18)	0.544
AIH simplified diagnosis score		7 (6, 8)	7 (6, 8)	0.044
Laboratory values				
PLT/ULN		0.53 ± 0.22	0.48 ± 0.18	0.097
ALT/ULN		4.54 (1.61, 9.54)	3.33 (1.06, 5.83)	0.214
AST/ULN		4.60 (1.57, 9.26)	3 (1.50, 8.68)	0.324
GGT/ULN		2.78 (1.51, 4.63)	2.22 (1.07, 3.84)	0.073
ALP/ULN		0.95 (0.73, 1.28)	0.89 (0.66, 1.24)	0.552
Albumin/ULN		0.73 (0.66, 0.80)	0.77 (0.69, 0.83)	0.029
TBIL/ULN		1.28 (0.65, 3.91)	0.88 (0.60, 2.32)	0.149
FBG/ULN		0.86 (0.76, 1)	0.89 (0.74, 1.01)	0.921
TGs/ULN		0.75 (0.53, 1.13)	0.69 (0.54, 1.12)	0.735
TC/ULN		0.71 ± 0.25	0.71 ± 0.23	0.994
Serum immunological features				
IgG/ULN		1.09 (0.85, 1.55)	1.29 (1.04, 1.62)	0.009
ANA +, n (%)		155 (93.4)	54 (96.4)	0.525
SMA +, n (%)		82 (49.4)	21 (37.5)	0.123
SLA/LP +, n (%)		9 (5.4)	2 (3.6)	0.734
Anti-LKM-1 +, n (%)		13 (7.8)	4 (7.1)	1
AMA +, n (%)		3 (1.8)	0	0.574
Histological features of diagnostic biopsy				
Interface hepatitis, n (%)				0.510
No significant hepatitis ^a^, n (%)		106 (63.9)	33 (58.9)	
Significant hepatitis ^a^, n (%)		60 (36.1)	23 (41.1)	
Plasma cells, n (%)		120 (72.3)	37 (67.3)	0.477
Hepatocyte rosetting, n (%)		42 (25.3)	12 (21.4)	0.559
Fibrosis, n (%)				<0.001
No significant fibrosis ^b^, n (%)		105 (63.3)	20 (35.7)	
Significant fibrosis ^b^, n (%)		61 (36.7)	36 (64.3)	

Data are presented as the medians (quartile 25, quartile 75)/means (± standard deviation) or numbers (proportion).

a No significant inflammation: A0-A2, significant inflammation: A3-A4.

b No significant fibrosis: F0-F2, Significant fibrosis: F3-F4.

ULN values: PLT 350×10^9^/L, ALT 35-50 U/L, AST 35-40 U/L, GGT 45-60 U/L, ALP 125-135 U/L, albumin 51-55 g/L, TBIL 20-23.9 μmol/L, FBG 6.1 mmol/L, TG 1.7-1.92 mmol/L, TC 5.18-5.7 mmol/L, IgG 16 g/L.

ULN upper limits of normal, AIH autoimmune hepatitis, BMI body mass index, CAP controlled attenuation parameter, PLT platelet, ALT alanine aminotransferase, AST aspartate aminotransferase, ALP alkaline phosphatase, GGT gamma-glutamyl transpeptidase, TBIL total bilirubin, FBG fasting blood glucose, TGs triglycerides, TC total cholesterol, IgG immunoglobulin G, ANA antinuclear antibody, SMA smooth muscle antibody, SLA/LP soluble liver antigen/liver pancreas antibody, LKM-1 liver-kidney microsomal-1 antibody, AMA antimitochondrial antibody.

### Treatment regimen and complete biochemical response

The complete biochemical response has been proven to be an excellent indicator of the response to AIH therapy. We investigated whether steatosis affects the biochemical response of patients. All patients underwent immunosuppressive therapy, and the treatment regimen is summarized in **Supplementary Table 1**. Patients were initially treated with prednisolone. During maintenance therapy, 60.8% of patients applied prednisolone monotherapy, and most (69.6%) achieved a complete biochemical response. A total of 37.4% combined with AZA, of which 65.1% obtained a complete biochemical response. Only four patients (< 2%) received MMF, and the biochemical response rate was low.

There was no significant difference in treatment regimens or prednisolone dosage taken by patients with different baseline hepatic steatosis statuses. Under immunosuppressive treatment, 73 patients showed an insufficient biochemical response. Patients with hepatic steatosis presented a significantly higher proportion of insufficient biochemical response compared with no steatosis patients (62.5% *vs*. 22.9%, P < 0.001). For insufficient biochemical response, there was no difference in the persistent elevations of transaminases and IgG between AIH with or without hepatic steatosis (**Supplementary Table 2**). In the univariate logistic regression analysis, hepatic steatosis (OR: 5.61, 95% CI: 2.93 to 10.77, P < 0.001) was associated with an insufficient biochemical response. After adjusting for potential confounders based on prior knowledge (Model 1 adjusted for sex, age, BMI, arterial hypertension, diabetes mellitus, and FBG; Model 2 further adjusted for ALT, AST, IgG, cirrhosis, fibrosis of biopsy, interface hepatitis, biochemical response, and corticosteroid dose) by a multivariable regression model, hepatic steatosis was found to independently predict biochemical response (P < 0.001 of all models) (**Table 3**). Significant steatosis was also an independent risk factor for insufficient biochemical response (**Table 3**).

**Table 3:** Univariable and multivariable logistic regression analysis for the impact of hepatic steatosis on the risk of insufficient biochemical response.

Variables	Unadjusted		Adjusted Model 1 ^a^		Adjusted Model 2 ^b^	
	OR (95% CI)	*P*	OR (95% CI)	*P*	OR (95% CI)	*P*
Female gender	1.12 (0.46, 2.70)	0.807				
Age at diagnosis, y	1 (0.98, 1.02)	0.875				
BMI (kg/m^2^)	1.06 (0.96, 1.16)	0.264				
CAP (dB/m)	1.01 (1, 1.02)	<0.001	1.02 (1, 1.02)	0.005	1.02 (1.01, 1.03)	0.001
Steatosis	5.61(2.93, 10.77)	<0.001	7.4 (2.99, 18.29)	<0.001	8.07 (2.67, 24.41)	<0.001
Significant steatosis ^c^	7.45(2.59, 21.44)	<0.001	7.62(2.63, 22.05)	<0.001	10.66 (2.67, 42.57)	<0.001
Arterial hypertension	1.55 (0.78, 3.06)	0.208				
Diabetes mellitus	2.93 (1.35, 6.34)	0.006				
Extrahepatic autoimmune disease	0.83 (0.40, 1.69)	0.599	0.95 (0.42, 2.15)	0.894	1.25 (0.46, 3.38)	0.659
Cirrhosis	2.25 (1.19, 4.25)	0.012	2.1 (0.95, 4.67)	0.067		
PLT/ULN	0.56 (0.14, 2.18)	0.4	0.29 (0.06, 1.49)	0.139	1 (0.16, 6.21)	0.994
ALT/ULN	0.99 (0.96, 1.03)	0.723	0.98 (0.95, 1.02)	0.408		
AST/ULN	1.02 (0.98, 1.05)	0.332	1.01 (0.98, 1.06)	0.49		
ALP/ULN	1.42 (0.85, 2.37)	0.177	1.29 (0.74, 2.24)	0.371	1.15 (0.56, 2.35)	0.7
Albumin/ULN	0.33 (0.02, 4.94)	0.424	0.02 (0, 0.54)	0.021	0.98 (0.35, 2.72)	0.97
TBIL/ULN	1 (0.92, 1.09)	0.998	0.99 (0.89, 1.10)	0.794	0.89 (0.75, 1.04)	0.139
FBG/ULN	2.07 (0.74, 5.8)	0.167				
IgG/ULN	4.68 (2.40, 9.15)	<0.001	6.33(2.86, 14.02)	<0.001		
Significant interface hepatitis ^d^	1.63 (0.92, 2.90)	0.093	2.18 (1.09, 4.37)	0.028		
Hepatocyte resetting	1.28 (0.67, 2.42)	0.456	1.40 (0.67, 2.93)	0.377	1.63 (0.66, 4.01)	0.289
Plasma cells	0.62 (0.34, 1.14)	0.127	0.88 (0.44, 1.78)	0.728	0.64 (0.28, 1.47)	0.293
Significant fibrosis ^e^	2.32 (1.31, 4.1)	0.04	3 (1.5, 5.99)	0.002		
High dose prednisolone ^f^	1.08 (0.61, 1.9)	0.796	0.71 (0.38, 1.39)	0.314		
Prednisolone + others (AZA, MMF) *vs.* prednisolone only	1.32 (0.75, 2.33)	0.340	1.62 (0.82, 3.19)	0.166	1.06 (0.48, 2.36)	0.886

a Model 1: adjustment for sex, age, BMI, arterial hypertension, diabetes mellitus, and FBG.

b Model 2: Model 1 + further adjustment for ALT, AST, IgG, cirrhosis, histological fibrosis, interfacial hepatitis, and corticosteroid dose.

c Significant steatosis: CAP ≥ 268 dB/m.

d Significant interfacial inflammation: A3-A4.

e Significant fibrosis: F3-F4.

f High-dose prednisolone defined as ≥ 30 mg prednisolone/day.

ULN values: ULN values: PLT 350×10^9^/L, ALT 35-50 U/L, AST 35-40 U/L, ALP 125-135 U/L, albumin 51-55 g/L, TBIL 20-23.9 μmol/L, FBG 6.1 mmol/L, IgG 16 g/L.

ULN upper limits of normal, AIH autoimmune hepatitis, OR odds ratio, CI confidence interval, BMI body mass index, CAP controlled attenuation parameter, PLT platelet, ALT alanine aminotransferase, AST aspartate aminotransferase, ALP alkaline phosphatase, TBIL total bilirubin, FBG fasting blood glucose, IgG immunoglobulin G, AZA azathioprine, MMF mycophenolate mofetil.

Furthermore, sensitivity analysis was conducted to assess the effect of different degrees of hepatic steatosis on the risk of insufficient biochemical response and the predictive power of hepatic steatosis on the insufficient biochemical response at different degrees of fibrosis and different doses of corticosteroids. It was found that any degree of hepatic steatosis was independently associated with insufficient biochemical response. Subgroup analysis showed that hepatic steatosis remained associated with insufficient biochemical response in patients regardless of different degrees of fibrosis and doses of prednisolone, both in adjustment Models 1 and 2 (**Supplementary Table 3**). Hepatic steatosis had better stability in predicting an insufficient biochemical response. We further found that 18 (24.7%) patients were identified as nonresponsive to biochemical treatment at the 4-week follow-up. The presence of hepatic steatosis resulted in a lesser decrease in ALT and AST, but there was no significant difference compared to the group without steatosis (**Supplementary Figure 2**).

### Follow-up of LS

**Figure 2:**
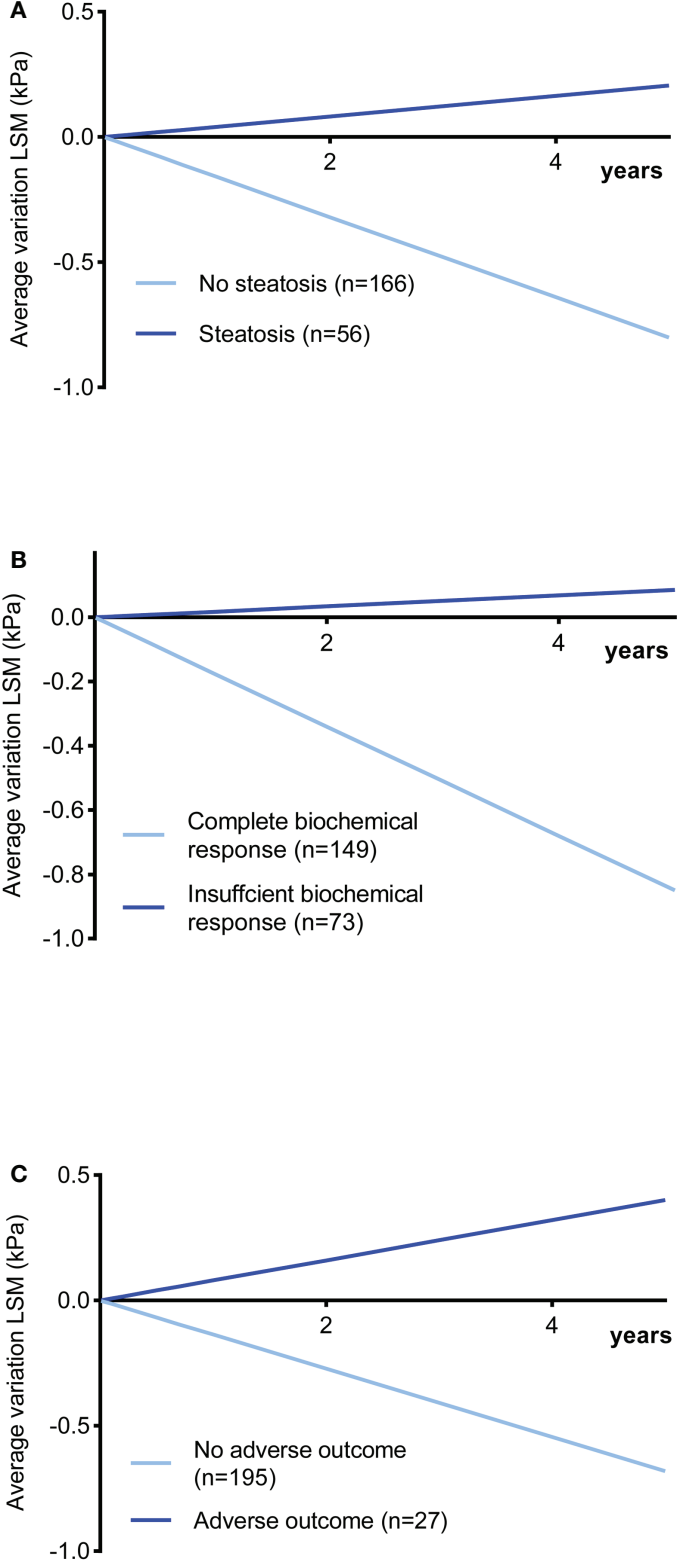
Changes in LSM in kPa as a function of time in AIH patients during follow-up (slope: changes in LS per year as percentages from baseline values). **(A)** With steatosis *vs.* without steatosis (P < 0.001). **(B)** Insufficient response *vs.* complete biochemical response (P < 0.001). **(C)** Poor outcome *vs.* no poor outcome (P < 0.001). LSM liver stiffness measurement.

The insufficient biochemical response might contribute to the progression of the disease, especially the extent of fibrosis. LS was validated with reliable accuracy and reproducibility to assess liver fibrosis as a noninvasive tool in AIH (10). Thus, we aimed to investigate the effect of steatosis on fibrosis development by following up on LS. Two hundred twenty-two patients underwent 761 TE with a median follow-up time of 3 years (range 1-9 years). The entire cohort had a decrease in LS of 9.6%/year (95% CI: -11.1% to -9.1%), including 61 (27.5%) patients increasing and 161 (72.5%) decreasing. The trends of △LS% as a function of time are shown in **Figure 2**. Compared with the significant decrease without steatosis (-16%/year, 95% CI: -19.3% to -12.6%, P < 0.001), a significant increase in LS was observed in patients with steatosis (4.1%/year, 95% CI: 1.5% to 6.7%, P = 0.002). Moreover, patients with insufficient biochemical response also showed a considerable increase in LS (1.7%/year, 95% CI: -1.8% to 5.2%, P = 0.339) compared with the complete biochemical response group (-17.1%/year, 95% CI: -20.4% to -13.7%, P < 0.001). Notably, people with adverse prognostic outcomes showed the most significant increases in LS (8%/year, 95% CI: 0.4% to 15.7%, P = 0.041). The relationship between annual changes in LSM and hepatic steatosis in the first three years was analysed in detail in **Supplementary Table 4**.

### Long-term outcome

**Figure 3:**
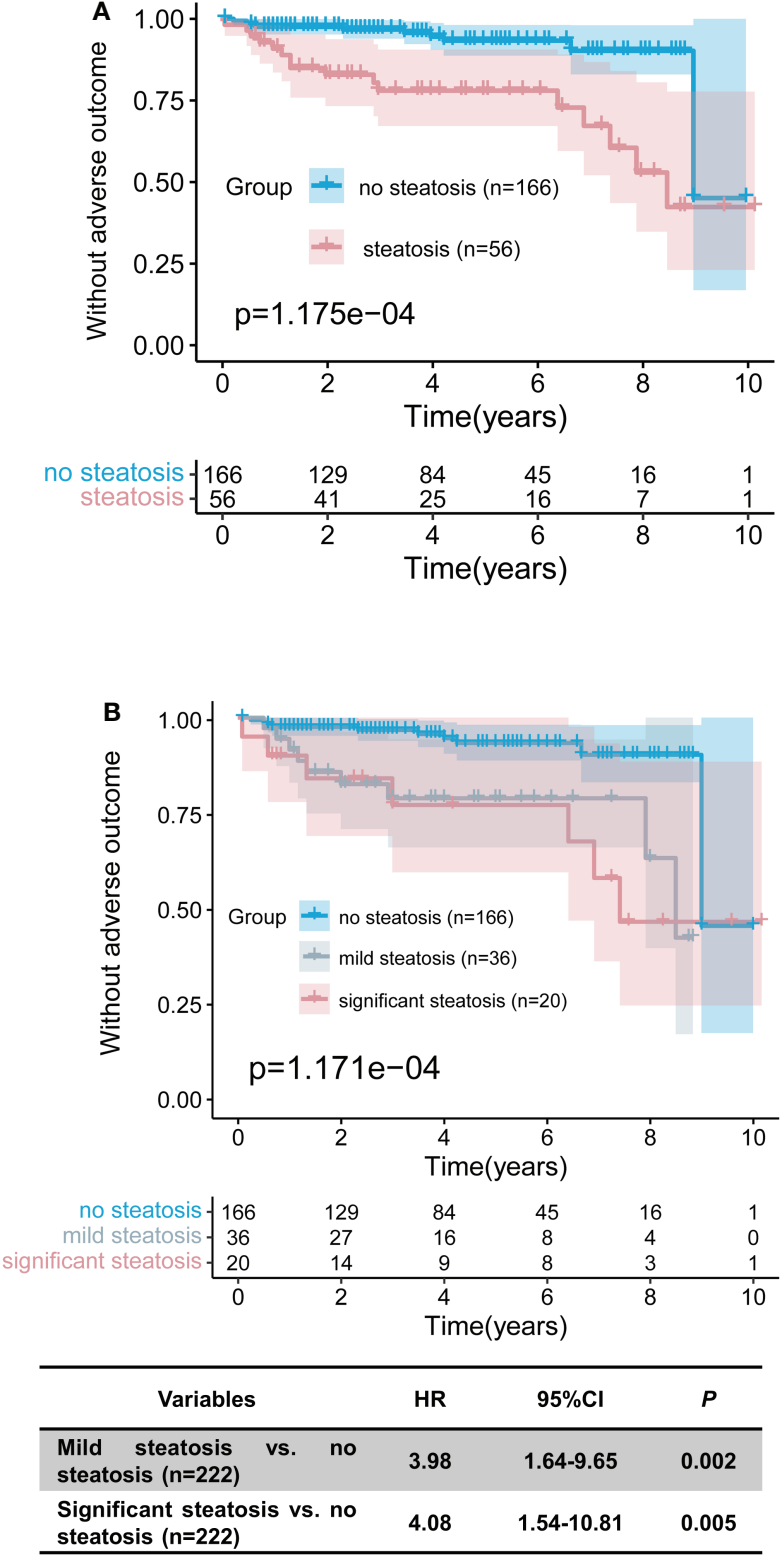
Kaplan‐Meier plots predict the association between the adverse outcome-free probability and hepatic steatosis. **(A)** Between patients with and without steatosis. **(B)** Stratified analysis of different degrees of hepatic steatosis. Cumulative, AIH autoimmune hepatitis, HR hazard ratio, CI confidence interval.

As stated above, steatosis might accelerate fibrosis progression; thus, we further analysed the effect on long-term adverse outcomes. The median follow-up was 48 months (range 7-128 months), with a similar time in the steatosis group and the no steatosis group (45.5 *vs.* 48, P = 0.602). During follow-up, 27 (12.2%) patients suffered adverse outcomes (three cirrhosis onset, six ascites, 12 variceal bleeding, four with both ascites and variceal bleeding, and two liver-related death). The probability of 5-year adverse outcome-free survival was 91.4% for patients without steatosis (95.2%) and with steatosis (80.4%) (P = 0.001). In univariate Cox analysis, the presence of hepatic steatosis before treatment was closely related to adverse outcomes (**Table 4**). Similarly, log-rank analysis depicted that the steatosis group had a significantly higher adverse outcome probability by the Kaplan‐Meier plots (P < 0.001; **Figure 3A**). In addition, further multivariate-adjusted analysis identified hepatic steatosis as an independent predictor of unsatisfactory outcomes in two models (Model 1 adjusted for sex, age, BMI, arterial hypertension, diabetes mellitus, and FBG; Model 2 further adjusted for ALT, AST, IgG, cirrhosis, fibrosis of biopsy, interface hepatitis, biochemical response, and corticosteroid dose) (**Table 4**). Furthermore, significant steatosis was also an independent risk factor for unsatisfactory outcomes (**Table 4**).

**Table 4:** Univariable and multivariable logistic regression analyses revealed the impact of hepatic steatosis on the risk of adverse outcomes.

Variables	Unadjusted		Adjusted Model 1 ^a^		Adjusted Model 2 ^b^	
	HR (95% CI)	*P*	HR (95% CI)	*P*	HR (95% CI)	*P*
Female gender	0.23 (0.1, 0.54)	0.001				
Age at diagnosis, y	0.99 (0.96, 1.02)	0.269				
BMI (kg/m^2^)	1.05 (0.93, 1.2)	0.43				
CAP (dB/m)	1.01 (1, 1.02)	0.007	1.01 (1, 1.02)	0.094	1.01 (1, 1.02)	0.231
Steatosis	4.02 (1.86, 8.69)	<0.001	4.71(1.64, 13.55)	0.004	4.07(1.04, 15.89)	0.043
Significant steatosis ^c^	4.23 (1.92, 8.83)	<0.001	4.82(1.84, 12.62)	0.001	4.49(1.48, 13.58)	0.008
Arterial hypertension	1.49 (0.63, 3.53)	0.371				
Diabetes mellitus	1.66 (0.69, 3.98)	0.254				
Extrahepatic autoimmune disease	0.97 (0.37, 2.59)	0.957	1.44 (0.5, 4.1)	0.5	1.03 (0.27, 4.01)	0.966
Cirrhosis	2.03 (0.94, 4.37)	0.07	2.84 (1.04, 7.75)	0.042		
PLT/ULN	0.75 (1.12, 4.82)	0.764	0.36 (0.37, 3.46)	0.373	1 (0.1, 10.06)	0.996
ALT/ULN	1.03 (1, 1.07)	0.089	1 (0.95, 1.05)	0.92		
AST/ULN	1.02 (0.97, 1.06)	0.428	1 (0.95, 1.07)	0.816		
ALP/ULN	1.7 (0.92, 3.16)	0.093	1.37 (0.69, 2.74)	0.371	1.53 (0.62, 3.78)	0.36
Albumin/ULN	0.51 (0.01, 21)	0.72	0.25 (0, 25.74)	0.557	1.08 (0.34, 3.46)	0.9
TBIL/ULN	0.98 (0.85, 1.12)	0.746	0.88 (0.69, 1.12)	0.285	0.8 (0.56, 1.13)	0.199
FBG/ULN	1.31 (0.3, 5.71)	0.721				
IgG/ULN	0.97 (0.61, 1.55)	0.909	0.93 (0.56, 1.55)	0.78		
Significant interface hepatitis ^d^	1.91 (0.9, 4.06)	0.094	2.06 (0.83, 5.1)	0.118		
Hepatocyte resetting	0.94 (0.38, 2.34)	0.892	0.73 (0.24, 2.27)	0.592	0.95 (0.29, 3.17)	0.939
Plasma cells	2.32 (0.87, 6.16)	0.091	2.18 (0.71, 6.73)	0.176	2.56 (0.8, 8.2)	0.113
Significant fibrosis ^e^	2.15 (0.96, 4.83)	0.065	3.33 (1.19, 9.33)	0.022		
Insufficient biochemical response	3.11 (1.42, 6.82)	0.005	2.52 (1.02, 6.2)	0.045		
High dose prednisolone ^f^	0.46 (0.21, 1.01)	0.052	0.28 (0.1, 0.78)	0.015		
Prednisolone only *vs.* prednisolone + others (AZA, MMF)	0.89 (0.4, 1.97)	0.776	1.73 (0.66, 4.52)	0.263	1.35 (0.49, 3.74)	0.567

a Model 1: adjustment for sex, age, BMI, arterial hypertension diabetes mellitus and FBG.

b Model 2: Model 1 + further adjustment for ALT, AST, IgG, cirrhosis, histological fibrosis, interfacial hepatitis, corticosteroid dose and biochemical response.

c Significant steatosis: CAP ≥ 268 dB/m.

d Significant interface inflammation: A3-A4.

e Significant fibrosis: F3-F4.

f High-dose prednisolone defined as ≥ 30 mg prednisolone/day.

ULN values: ULN values: PLT 350×10^9^/L, ALT 35-50 U/L, AST 35-40 U/L, ALP 125-135 U/L, albumin 51-55 g/L, TBIL 20-23.9 μmol/L, FBG 6.1 mmol/L, IgG 16 g/L.

ULN upper limits of normal, AIH autoimmune hepatitis, HR hazard ratio, CI confidence interval, BMI body mass index, CAP controlled attenuation parameter, PLT platelet, ALT alanine aminotransferase, AST aspartate aminotransferase, ALP alkaline phosphatase, TBIL total bilirubin, FBG fasting blood glucose, IgG immunoglobulin G, AZA azathioprine, MMF mycophenolate mofetil.

Next, stratified analysis was conducted based on the absence of steatosis, mild steatosis, or significant steatosis. Univariate Cox regression analysis found that any degree of steatosis was associated with poor outcomes (**Supplementary Figure 3**). Patients with significant steatosis had the highest risk of adverse outcomes, followed by mild steatosis and the lowest in the no steatosis group (**Figure 3B**). Further multivariate sensitivity analysis explored the stability of steatosis to predict adverse prognosis. Although significant steatosis was an independent adverse prognostic factor, the increased degrees of steatosis did not independently aggravate the adverse prognosis, possibly due to the small number of adverse events for the different stratifications of steatosis. In addition, among patients with different degrees of fibrosis, hepatic steatosis showed an increased risk of adverse outcomes for patients without significant fibrosis. For patients on low-dose prednisolone, baseline hepatic steatosis remained independently associated with poor outcomes (**Supplementary Table 5**).

## Discussion

Our results are of particular clinical relevance because data on the reliability of the effect on hepatic steatosis coincident with AIH have thus far been missing. AIH is a rare disease with significant heterogeneity of the clinical spectrum and is easily misdiagnosed, leading to cirrhosis progression, liver transplantation, and death (30). Concomitant with hepatic steatosis increases the difficulty of diagnosis, resulting in the rapid progression of AIH. However, it has not received enough attention in clinical practice. In this multicentre cohort study, steatosis was potentially associated with fibrosis progression and was an independent risk factor for insufficient biochemistry remission and adverse long-term outcomes.

Steatosis in hepatocytes enhances vulnerability to other damaging factors (31), which could promote the generation of reactive oxygen species (ROS), tumour necrosis factor-alpha (TNF-α), interleukin 6 (IL-6), interleukin 1 (IL-1), and plasminogen activator inhibitor-1 (PAI-1) to further activate signalling pathways and increase susceptibility to genetic polymorphisms (32–35). It is well reported that steatosis can be an essential cofactor accelerating the progression of liver damage in chronic hepatitis C patients (36). However, there remains a lack of data regarding the effects of hepatic steatosis coincident with AIH. In Western countries, a study of 73 AIH patients suggested that coexisting AIH and NASH were more likely to lead to adverse clinical outcomes than were AIH-only patients. It should be noted that this was a small retrospective study, and the conclusions cannot be drawn safely (8). On the other hand, the IAIHG designed a large multicentre retrospective study and showed that AIH patients with NAFLD were more likely to decompensate during follow-up. Histologically, significant fibrosis, portal inflammation, and plasma cell infiltration were observed more frequently in NAFLD/AIH compared to those with NAFLD alone (37). However, there is no evidence that NAFLD/NASH is an aggravating factor to the overall prognosis (37). The low number of AIH patients with concurrent NASH (19/583, 3.3%) could be responsible for these favourable outcomes. Relying on liver biopsy to diagnose NASH might be subject to sampling error, increasing false-negatives. Better methods must be applied to identify NAFLD/NASH. Our study enrolled a relatively large cohort from multiple medical centres and used CAP to quantify the extent of steatosis infiltration. Previous and present studies have confirmed that CAP is a superior quantifier for most steatosis infiltration pathologically (15, 17). This study provides a comprehensive interpretation of the effect of steatosis infiltration on AIH. At baseline, our study demonstrated that steatosis was detected more frequently in diabetes and obesity with histologically progressive fibrosis at diagnosis, consistent with previous studies. Thus, further blood pressure and glucose control might be needed to avoid steatosis in AIH patients.

In addition, a recent study strongly indicated that a complete biochemical response might better reflect a stable suppression of disease activity over the long term, thereby reducing the need for a follow-up liver biopsy (20). Therefore, our study estimated the response of hepatic steatosis to biochemical response, demonstrating that steatosis is an independent and stable risk factor for predicting complete biochemical response after adjusting for confounders. We then performed a sensitivity analysis and found that any degree of steatosis was strongly associated with insufficient biochemical remission. Studies have shown that a high dose of prednisone may correlate with the response to treatment by reducing hepatic inflammation (38). However, prednisone can exacerbate disturbances in lipid metabolism. There is a lack of recommendations regarding prednisone dose in patients with combined steatosis. In addition, studies have shown that patients with significant fibrosis are less likely to undergo a biochemical response (39). The metabolism of corticosteroids is severely impaired in patients with extensive fibrosis, which contributes to diminished efficacy (40). Therefore, to eliminate the influence of these factors, we performed a subgroup analysis. Our study found that steatosis remained an independent predictor of biochemical response insufficiency, including patients on high/low dose prednisone and significant/no-significant fibrosis.

Steatosis has also been proven to be an important motivating factor for liver fibrosis from periportal to bridging fibrosis and eventually cirrhotic remodelling with liver failure and, finally, hepatocarcinogenesis (41). Our study used TE to follow up on liver stiffness changes in patients with steatosis. TE has been certified as an objective and robust tool to monitor disease progression and avoid serial biopsies and is recommended by the AASLD practice guidance of AIH (10). In our study, we recorded LS approximately six months after treatment. We used variations in LS to reflect fibrosis changes to avoid a potential bias due to hepatic inflammation. Moreover, the increase in LS% mainly occurred in the group with hepatic steatosis. Our results might assist in improving treatment monitoring in AIH patients.

We further analysed the effect of hepatic steatosis on long-term prognosis. There are no therapeutic practice guidelines for hepatic steatosis in AIH patients, and the standard AIH management is initially corticosteroids and follow-up maintenance (42). There was no statistically significant difference between hepatic steatosis and nonsteatosis patients concerning the therapy method. The multivariate analysis identified the presence of steatosis at accession as an independent predictor of unsatisfactory outcomes. A combination of steatosis is responsive to corticosteroid therapy, but chronic inflammation and immune system disturbance might influence the long-term stable response to corticosteroids. The predictive effect of steatosis on poor prognosis was diminished when corticosteroids were administered at high doses, which might be due to the potent suppression of liver inflammation and the rapid attainment of biochemical remission. When included in patients with significant fibrosis, fibrosis might play a decisive role in poor prognosis outweighing the effect of steatosis. Perhaps closer lifestyle modifications, better suppression of inflammation, and progressive fibrosis reduction are of significant importance.

There were three limitations of this study. First, although we innovatively used noninvasive measures to assess efficacy, it is best to follow up with regular histological data for verification. However, biopsies are invasive procedures performed regularly for a relatively large cohort of patients and seem impractical. Another major limitation is that the 95% CI range is wide due to the relatively small availability of detected events, particularly when performing sensitivity analysis. In addition, this study was retrospective, perhaps with an inherent selection bias. The mechanism of the impact on the outcome and treatment response of steatosis in well-established AIH cases is largely unknown. In the future, the discovery of a specific mechanism to reveal how hepatic steatosis interferes with the AIH response to therapy is expected.

## Conclusion

In summary, our study found that hepatic steatosis, as measured by CAP, might be related to the apparent progression of liver fibrosis and was significantly and independently associated with a higher risk of insufficient biochemistry response and adverse outcome prognosis. For AIH patients, it is essential to actively maintain a reasonable weight and control diabetes, hypertension, and hyperlipidaemia. During treatment, the dosage of corticosteroids should be considered. Individualized management and strict follow-up of AIH patients with hepatic steatosis should be carried out.

## Data availability statement

The original contributions presented in the study are included in the article/**Supplementary Material**. Further inquiries can be directed to the corresponding authors.

## Ethics statement

The studies involving human participants were reviewed and approved by the Ethics Committees of three participating centers. Written informed consent to participate in this study was provided by the participants’ legal guardian/next of kin.

## Author contributions

Guarantor of the article: BW and JL. Study concept and design: PL, ML, BW and JL. Drafting manuscript: PL and ML. Statistical analysis: PL and ML. Data collection: PL, ML, LLZ, HY,CZ, JC, RS, LZ, and QZ. The revision of the manuscript: JL and BW. All authors contributed to the article and approved the submitted version.
