# Supplementary material for: Impact of hepatic steatosis on treatment response of autoimmune hepatitis: A retrospective multicentre analysis
Source: Front Immunol. 2022 Dec 14;13:1040029. doi: 10.3389/fimmu.2022.1040029 (PMC9795183; doi:10.3389/fimmu.2022.1040029)
Supplement: Supplementary file 1 [file DataSheet_1.docx]

Supplementary Material

## Supplementary Figures


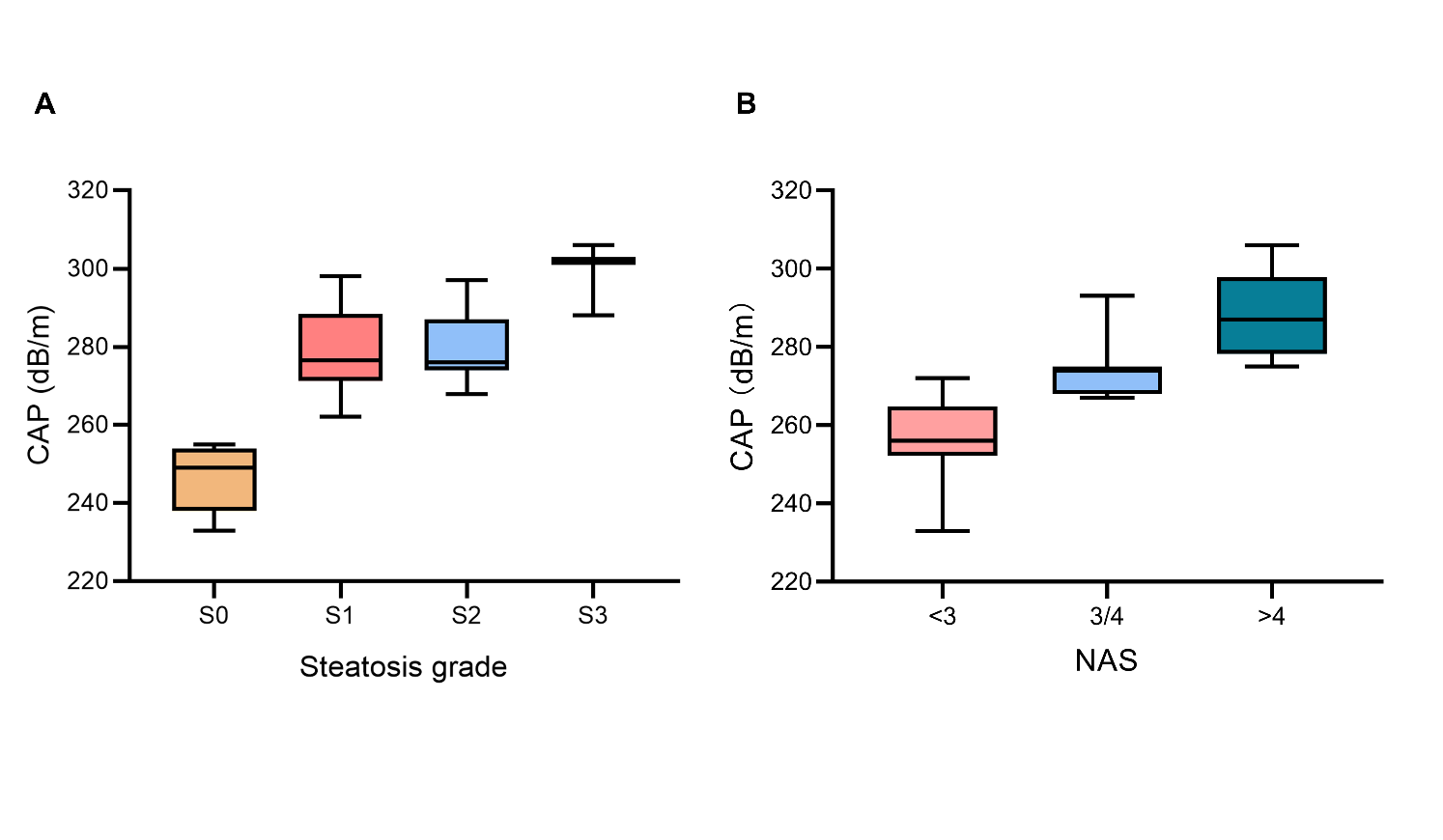


**Supplementary Figure 1.** Boxplot of **(A)** CAP vs steatosis grade, **(B)** CAP vs NAS. **(A)** CAP values increase with increasing steatosis grade (Kruskal-Wallis test P < 10^–4^). **(B)** CAP values increase with increasing NAS (Kruskal-Wallis test P < 10^–4^). Abbreviations: CAP controlled attenuation parameter, NAS nonalcoholic fatty liver disease activity scores.

**
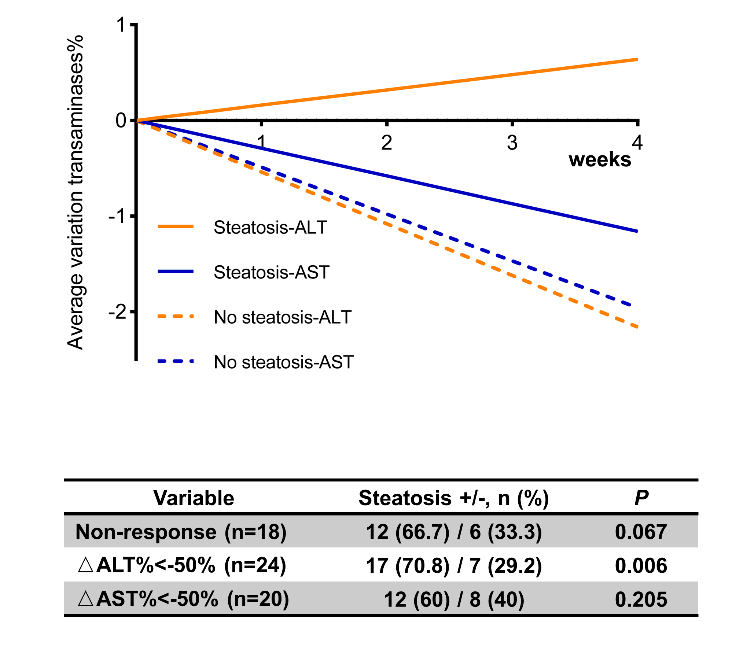
**

**Supplementary Figure 2.** Changes of transaminases (ALT/AST) in 4 weeks after initiation of treatment (slope: changes of ALT/AST at the fourth week as percentages from baseline values). △ALT% = (ALT at 4th week – ALT at baseline)/ALT at baseline. △AST% = (AST at fourth week – AST at baseline)/AST at baseline. Non-response: <50% decrease of serum transaminases within four weeks after initiation of treatment. Abbreviations: ALT alanine aminotransferase, AST aspartate aminotransferase

**
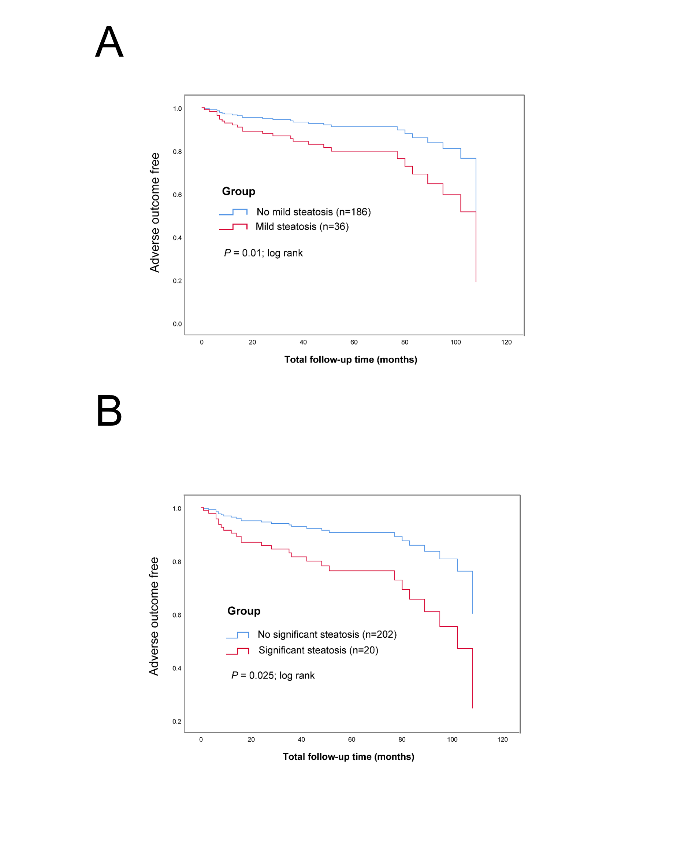
**

**Supplementary Figure 3.** Cumulative adverse outcome-free survival stratified by different degrees of hepatic steatosis by log-rank test. **(A)** between with and without mild steatosis. **(B)** between with and without significant steatosis.

## Supplementary Tables

**Supplementary Table 1.** Treatment regimens of patients.

| Treatment regimens | Data (n=222) |
| --- | --- |
| *Initial treatment* |  |
| Prednisolone, n (%) | 222 (100) |
| Dose, mg | 30 (24, 40) |
| Low dose corticosteroid at initial treatment (defined as ＜30 mg prednisolone/day), n (%) | 97 (43.7) |
| High dose corticosteroid at initial treatment (defined as ≥ 30 mg prednisolone/day), n (%) | 125 (56.3) |
| *Maintenance therapy* |  |
| Prednisolone only, n (%) | 135 (60.8) |
| Prednisolone + AZA, n (%) | 83 (37.4) |
| Prednisolone + MMF, n (%) | 4 (1.8) |

Data are presented as median (quartile 25, quartile 75) or number (proportion).

Abbreviations: AZA azathioprine, MMF mycophenolate mofetil.

**Supplementary Table 2.** Profiles of transaminases and IgG in patients with insufficient biochemical response.

|  | Only transaminases elevation | Only IgG elevation | Both transaminases and IgG elevations |
| --- | --- | --- | --- |
| AIH only, n (%) | 5 (13.2) | 17 (44.7) | 16 (42.1) |
| AIH with steatosis, n (%) | 4 (11.4) | 16 (45.7) | 15 (42.9) |

Data are presented as number (proportion).

Abbreviations: AIH autoimmune hepatitis.

**Supplementary Table 3.** Sensitivity analysis of the risk factors of insufficient biochemical response.

| Variables | Adjusted Model 1 ^a^ | |  | Adjusted Model 2 ^b^ | |
| --- | --- | --- | --- | --- | --- |
|  | OR (95%CI) | *P* |  | OR (95%CI) | *P* |
| Mild steatosis ^c^ *vs.* no steatosis (n=222) | 5.49 (1.98, 15.21) | 0.001 |  | 5.43 (1.6, 18.45) | 0.007 |
| Significant steatosis ^c^ *vs.* no steatosis (n=222) | 9.61 (2.73, 33.76) | < 0.001 |  | 15.19 (2.89, 79.92) | 0.001 |
| Significant steatosis ^c^ *vs.* no significant steatosis (n=222) | 5.35 (1.65, 17.32) | 0.005 |  | 7.75 (1.7, 35.24) | 0.008 |
| CAP when only patients without significant fibrosis ^d^ were included (n=125) | 9.08 (1.67, 49.37) | 0.011 |  | 28.51 (1.97, 413.66) | 0.014 |
| CAP when only patients with significant fibrosis ^d^ were included (n=97) | 5.3 (1.57, 17.91) | 0.007 |  | 5.57 (1.3, 23.97) | 0.021 |
| CAP when patients on low dose prednisolone ^e^ were included (n=125) | 24.48 (4.1, 146.25) | < 0.001 |  | 33.93 (3.15, 365.05) | 0.004 |
| CAP when patients on high dose prednisolone ^e^ were included (n=97) | 4.23 (1.33, 13.43) | 0.015 |  | 4.17 (1.06, 16.48) | 0.042 |

^ab^ Model 1/2 were fully adjusted for the same covariates as Model 1/2 in the Table 3.

^c^ Mild steatosis: CAP 248-267 dB/m, significant steatosis: CAP ≥ 268 dB/m.

^d^ Significant fibrosis: F3-F4.

^e^ Low dose prednisolone defined as ＜30 mg prednisolone/day and high dose prednisolone defined as ≥ 30 mg prednisolone/day.

Abbreviations: OR odds ratio, CI confidence interval, CAP controlled attenuation parameter, AZA azathioprine, MMF mycophenolate mofetil.

**Supplementary Table 4.** The relationship between the positive rate of △LS% and steatosis, biochemical response, and long-time adverse outcome in the first three years with follow-up.

| Variables |  | 1st year △LS% + | *P* |  | 2nd year △LS% + | *P* |  | 3rd year △LS% + | *P* |
| --- | --- | --- | --- | --- | --- | --- | --- | --- | --- |
| Steatosis based on CAP, n (%) |  | 49 (87.5) | <0.001 |  | 33 (73.3) | <0.001 |  | 20 (60.6) | <0.001 |
| Insufficient biochemical response, n (%) |  | 54 (77) | <0.001 |  | 37 (61.7) | <0.001 |  | 20 (48.8) | <0.001 |
| Adverse outcome, n (%) |  | 21 (77.8) | <0.001 |  | 17 (85) | <0.001 |  | 11 (73.3) | <0.001 |

Data are presented as number (proportion).

1st year △LS% (n=220, positive rate=38.6%), 2nd year △LS% (n=182, positive rate=31.3%), 3rd year △LS% (n=137, positive rate=27.0%).

Abbreviations: LS liver stiffness, △LS the changes of LS, △LS% = (LS at year – LS at baseline)/ LS at baseline, + positive.

**Supplementary Table 5.** Sensitivity analysis of multivariate regression on the risk factors of adverse outcome.

| Variables | Adjusted Model 1 ^a^ | |  | Adjusted Model 2 ^b^ | |
| --- | --- | --- | --- | --- | --- |
|  | HR (95%CI) | *P* |  | HR (95%CI) | *P* |
| Significant steatosis ^c^ vs. no significant steatosis (n=222) | 2.53 (0.73, 8.79) | 0.143 |  | 1.88 (0.46, 8.57) | 0.357 |
| CAP when only patients without significant fibrosis ^d^ were included (n=125) | 2.85 (1.92, 42.34) | 0.447 |  | 15.3 (1.03, 40.04) | 0.049 |
| CAP when only patients with significant fibrosis ^d^ were included (n=97) | 3.21 (0.87, 11.87) | 0.08 |  | 3.51 (0.61, 20.38) | 0.161 |
| CAP when patients on low dose prednisolone ^e^ were included (n=125) | 8.12 (1.88, 34.97) | 0.005 |  | 13.73 (1.22, 154.25) | 0.034 |
| CAP when patients on high dose prednisolone ^e^ were included (n=97) | 1.62 (0.2, 13.22) | 0.651 |  | 2.79 (0.19, 41.74) | 0.457 |

^ab^ Model 1/2 were fully adjusted for the same covariates as Model 1/2 in the Table 3.

^c^ Significant steatosis: CAP ≥ 268 dB/m.

^d^ Significant fibrosis: F3-F4.

^e^ Low dose prednisolone defined as ＜30 mg prednisolone/day and high dose prednisolone defined as ≥ 30 mg prednisolone/day.

Abbreviations: HR hazard ratio, CI confidence interval, CAP controlled attenuation parameter, AZA azathioprine, MMF mycophenolate mofetil.
